# Deep hashing for global registration of preoperative CT and video images for laparoscopic liver surgery

**DOI:** 10.1007/s11548-025-03418-w

**Published:** 2025-05-23

**Authors:** Hanyuan Zhang, Sandun Bulathsinhala, Brian R. Davidson, Matthew J. Clarkson, João Ramalhinho

**Affiliations:** 1 UCL Hawkes Institute, Department of Medical Physics and Biomedical Engineering, UCL, UK; 2 Division of Surgery and Interventional Science, UCL, UK

**Keywords:** Laparoscopic Liver Surgery, Augmented Reality, Image Registration, Deep Hashing

## Abstract

****Purpose**:**

Registration of computed tomography (CT) to laparoscopic video images is vital to enable augmented reality (AR), a technology that holds the promise of minimising the risk of complications during laparoscopic liver surgery. Although several solutions have been presented in the literature, they always rely on an accurate initialisation of the registration that is either obtained manually or automatically estimated on very specific views of the liver. These limitations pose a challenge to the clinical translation of AR.

****Methods**:**

We propose the use of a content-based image retrieval (CBIR) framework to obtain an automatic robust initialisation to the registration. Instead of directly registering video and CT, we render a dense set of possible views of the liver from CT and extract liver contour features. To reduce feature maps to lower dimension vectors, we use a deep hashing (DH) network that is trained in a triplet scheme. Registration is obtained by matching the intra-operative image hashing encoding to the closest encodings found in the pre-operative renderings.

****Results**:**

We validate our method on synthetic and real data from a phantom and real patient data from eight surgeries. Phantom experiments show that registration errors acceptable for an initial registration are obtained if sufficient pre-operative solutions are considered. In seven out of eight patients, the method is able to obtain a clinically relevant alignment.

****Conclusion**:**

We present the first work to adapt DH to the CT to video registration problem. Our results indicate that this framework can effectively replace manual initialisations in multiple views, potentially increasing the translation of these techniques.

**Supplementary Information:**

The online version contains supplementary material available at 10.1007/s11548-025-03418-w.

## Introduction

Augmented reality (AR) is currently seen as an image-guidance solution that can mitigate the risk of laparoscopic liver surgery and therefore increase its uptake [1]. By projecting an overlay of the internal liver anatomy derived from a pre-operative image such as computed tomography (CT) onto the video showing the liver, surgeons can have vital information on the location of major blood vessels and tumours for increased safety [2]. The main challenge in AR deployment is the registration between the liver surface and the intra-operative laparoscopic video [3]. Compared to other registration problems, CT to video registration of the liver is particularly under-constrained due to the presence of insufflation induced deformations and the fact that video only captures a relatively small and partial view of the liver. Therefore, an accurate rigid initialisation is usually needed to guarantee that further deformable updates are clinically valid. Currently, this initialisation is obtained either manually [1] or automatically under the assumption that the video view captures enough liver features without occlusions. Therefore, AR translation would benefit from fast automatic global registration techniques that are reliable from less constrained views of the liver.

### Background

Video to CT registration was initially attempted through a 3D–3D formulation where the intra-operative surface of the liver is reconstructed from video using laser range scanners [4], intra-operative CT [5] and stereo laparoscopes [6, 7]. From these options, only stereo laparoscopes are compatible with the current scenario of laparoscopic surgery. Using stereo reconstructed surfaces, global registration has been obtained by matching surfaces with the globally optimal iterative closest point algorithm (Go-ICP) [7], by matching surfaces from the liver and its anterior ridge [6], and by matching keypoints through point correspondence-based deep neural networks [8, 9]. However, stereo reconstructed surfaces are not always robust across the camera viewing direction, and recently developed monocular depth estimation methods do not provide a reliable reconstruction as well.

Recently, this problem has been approached from a 2D-3D formulation where liver contours detected in the 2D video are used as features to projectively align the 3D surface. Initially, this approach was introduced by Koo et al. to obtain a deformable registration with a single monocular image [10] and later refined to include multiple views [11]. Since these works relied on a manual initialisation, further methods were introduced to estimate a starting global rigid alignment using a perspective-n-point (PnP) pose estimation within a random sample consensus (RANSAC) framework [12, 13]. To avoid heavy sampling and slower optimisation schemes from these methods, more recent deep learning techniques have been used to learn a patient-specific model in a self-supervised fashion to quickly estimate not only camera pose but also deformation in a joint framework [14, 15]. Despite showing promising results, these methods are most commonly tested on views that capture the whole liver shape with the falciform ligament removed, displaying a well-defined set of labelled contours. In many cases where the ligament is not surgically removed, only partial views with a limited set of contours can be visualised.

In order to obtain a fast initial alignment on partial views, we propose the use of a deep hashing [16] framework for registration that also follows a self-supervised scheme and does not require paired data for training [17]. In this framework, instead of optimising the registration, we treat it as a content-based image retrieval (CBIR) problem. Firstly, we pre-operatively simulate a large database of camera views from CT and encode each of them to a latent/hash representation. Intra-operatively, we encode the segmented intra-operative image contours and perform a fast nearest neighbour search to find the closest database hash codes and therefore most likely camera pose to represent the CT to video registration. To encode 2D images to meaningful codes, we extract commonly used liver contours and train a DH model using a triplet scheme [18] to learn to separate inaccurately and accurately registered poses [17]. Previously, hashing has been used for camera pose estimation [19] in liver surgery, but the hash codes were not learnable and required the use of a depth camera. Our main contributions are:

1. The first DH approach for the registration between 2D video and 3D CT in laparoscopic surgery.
2. An augmentation scheme to simulate occlusions and deformations during training.
3. A validation on retrospective patient cases where AR was deployed with a manual registration.

## Methods

**Fig. 1:**
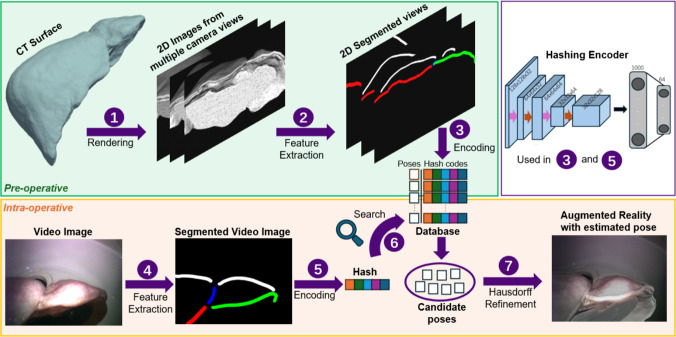
Pipeline for deep hashing-based registration. Pre-operatively, a trained hashing model is used to create codes for a set of possible rendered views from CT. Intra-operatively, the video is segmented, encoded and compared with the database to find the most likely render to represent the scene

Our registration pipeline is illustrated in Fig. 1. Pre-operatively, we first render a dense set of plausible views from which the liver can be observed (**1**). Then, we extract relevant liver features for registration (**2**) and encode them with a convolutional neural network (CNN)-based encoder to create a database that matches camera pose to hash code (**3**). Intra-operatively, video images are segmented for the same features extracted in the database generation step (**4**), encoded with the same model (**5**), and a nearest neighbour search is used to find the closest hash codes in Euclidean space (**6**). Finally, a refinement based on Hausdorff distance is applied to estimate the final camera pose and provide an AR overlay (**7**). Clinically, the objective of this method is to allow surgeons to quickly obtain an updated AR overlay display from any desired laparoscopic view during surgery. This rigid registration result can then be further refined with deformable registration methods [15].

### Pose generation and rendering

The first key component of our method is the definition of a space of solutions to be rendered and used in the patient-specific hash database. We define a database *F* as a set of camera poses with six degrees of freedom (three translations and three rotations) and a corresponding hash code. To define the set of poses, we first create a set of possible camera positions by sampling a spherical coordinate system centred at the liver surface mesh centre. For each liver mesh, we visually adjust the range of angles from which it is plausible to view the liver (mainly capturing the anterior surface) and manually define a range with minimum, maximum and step values for each of the two spherical angles ($\theta =\{\theta _{min}:\delta \theta :\theta _{max}\}$ and $\phi =\{\phi _{min}:\delta \phi :\phi _{max}\}$) and radial distance from centre $r=\{r_{min}:\delta r:r_{max}\}$. A set of 3D positions is created from all discrete combinations of elements of these sets. To sample different orientations, we set three ranges for Euler angles ($\alpha = \{\alpha _{min} : \delta \alpha : \alpha _{max} \}, \beta = \{\beta _{min} : \delta \beta : \beta _{max} \}$ and $\gamma =\{\gamma _{min} : \delta \gamma : \gamma _{max} \})$, and apply all possible combinations of Euler rotations at every sampled position in space. To define a reference rotation at each position from which multiple rotations are applied, we point the camera principal axis (z axis) towards the liver surface centre and align the x and y axes with the coronal plane of the patient.

For every pose, we render a 2D view of the CT surface using open-source libraries [20]. Following existing works on 2D-3D registration in liver surgery [3], we extract four distinct contours from each rendering: the anterior ridge of the right lobe, the anterior ridge of the left lobe, the falciform ligament, and the silhouette separating liver from background (**2** and **4** in Fig. 1). To extract these features quickly and robustly, we manually draw surface objects representing the ridge contours and ligament and apply morphological operations and edge filters to extract the silhouette. For every pose in *F*, we then obtain an feature map with distinct integer values for each of the four contours.

### Deep hashing

DH refers to the use of deep neural networks to convert images into low-dimensional hash codes. The main purpose of hashing models is to encode images that are semantically similar with hash codes that are close in hash space and images that are semantically different with hash codes that are far away in hash space [16].

#### Deep hashing model

We encode contour feature maps using the DH model reported in [17]. This model consists of a Siamese network with three 2D CNN blocks followed by two fully connected layers to extract a hash vector *h* with length 64 (top right section of Fig. 1). In a retrieval-based registration task, there are no semantics to constrain hash code learning—instead, codes close in hash space should represent an accurate registration [17]. Therefore, we train the Siamese Network in a contrastive learning scheme where a triplet of images is input separately to each CNN block: the Query $\textrm{x}_q$, which is the image to be registered, the Positive $\textrm{x}_p$ that represents an accurate registration, and the Negative $\textrm{x}_n$ that represents an inaccurate registration. Our objective is to drive the query image latent hash $h_q$ towards the latent hash of the positive $h_p$ and away from the hash of the negative $h_n$ through the contrastive loss

1 $$\begin{aligned} L_{c} = \max (0, m + D(h_q, h_p) - D(h_q, h_n)), \end{aligned}$$

where $D(\cdot ,\cdot )$ is the Euclidean distance operator and *m* a margin value to prevent overflow in the distance $D(h_q, h_n)$.

To ensure that hash space learning is not purely contrastive, we enforce semantic meaning in the learnt hash codes by using autoencoding as an auxiliary task. A CNN decoder symmetrical to the encoder blocks is used to decode hash codes $h_q$, $h_p$, $h_n$ to images $\hat{\textrm{x}}_q$, $\hat{\textrm{x}}_p$, $\hat{\textrm{x}}_n$. For this autoencoding task, we use the weighted reconstruction loss:

2 $$\begin{aligned} L_r = \frac{1}{N} \sum (w_{xq} \Vert \hat{\textrm{x}}_q - \textrm{x}_q\Vert ^2 + w_{xp} \Vert \hat{\textrm{x}}_p - \textrm{x}_p\Vert ^2 + w_{xn} \Vert \hat{\textrm{x}}_n - \textrm{x}_n)\Vert ^2) \end{aligned}$$

where weights $w_{xq}$, $w_{xp}$, $w_{xn}$ represent the ratio of nonzero pixels in each image.

Finally, in order to prevent codes from diverging with $L_c$, we use a binarisation loss to approach code values to either 1 or -1 and centre them around 0:

3 $$\begin{aligned} L_{b} = \Vert |h_{q}| - \textbf{1} \Vert ^2 + \Vert |h_{p}| - \textbf{1} \Vert ^2 + \Vert |h_{n}| - \textbf{1} \Vert ^2 \end{aligned}$$

Our final loss then becomes a weighted sum of three losses:

4 $$\begin{aligned} L = w_c L_c + w_r L_r + w_b L_b \end{aligned}$$

#### Training and inference

We train our model with a patient-specific approach. Firstly, we generate a database of possible training poses $F_{train}$ that angularly cover the range of possible views during surgery. At every training iteration, we sample a triplet of images by setting the query $\textrm{x}_q$ as a random image in the set, the negative $\textrm{x}_n$ as any random image that differs from the query in position by 30 mm or by rotation in $30^\circ$, and the positive $\textrm{x}_p$ as an augmented version of $\textrm{x}_q$. As in [17], the augmentation in the positive sample attempts to simulate expected differences in the contours between pre-operative and intra-operative settings. To simulate outcomes of deformation in 2D contours in a simplistic fashion, we introduce affine shear distortions. Then, we simulate occlusions of contours with rectangular masks centred at contour pixels and segmentation errors by randomly removing contour pixels and applying erosions and dilations to contours.

Once the model is trained for a patient, we generate a database $F_{test}$ with a higher number of samples with the same translation and rotation ranges but smaller steps. We choose to use a smaller and less time-consuming training set as the model is expected to generalise to finer poses that are interpolated from the coarser training poses.

### Registration

Once a database $F_{test}$ is generated for a patient-specific CT, registration with an intra-operative video image is obtained by segmenting the same contours in the image, encoding them with the trained model, and finding the set of *k* closest hash codes in the database. We use a set of *k* top poses or candidates as the top closest pose is not guaranteed to result in an accurate registration due to intra-operative deformations, occlusions, and non-uniqueness of camera views. Following step **7** in Fig. 1, to find an accurate registration within the retrieved *k* poses, we use a weighted Hausdorff distance between the query image and resulting retrieved rendered images to find the pose that results in the most similar contour rendering $\hat{J}_k$:

5 $$\begin{aligned} \hat{J}_k = \underset{J_{1}...,J_{k}}{\textrm{arg }} {\textrm{min}} \sum _{f}^{4} w_{I_{f}}H(I_{f}, J_{kf}) \end{aligned}$$

Here, *H*(., .) is the Hausdorff distance operator, *I* the intra-operative contours, and $J_{k}$ the contours of the k-th closest hash. The subscript *f* indicates the four used features, and the submasks corresponding to each of them. Weights $w_{I_{f}}$ are the ratio of pixels in each contour over all nonzero pixels in *I*.

## Experiments

We validate our approach on three sets of data, *in silico* synthetic data generated from a liver phantom surface, real video images from the same phantom, and video and CT data from eight laparoscopic liver surgeries.

For all cases, we train our DH with a batch size of 32 for 50 epochs with a learning rate of $10^{-4}$ using the Adam optimiser on a NVIDIA RTX 4090. All our models were trained with $w_c=10$, $w_{r}=100$, $w_{r}=1$, and the margin *m* in $L_c$ was set to half of the hash code size, 32. For the positive input augmentation, we apply uniformly distributed shearing ranging from $-10^{\circ }$ to $10^{\circ }$, remove randomly up to 5% of nonzero contour pixels, randomly apply between 2 and 5 dilation or erosions operations (one or the other with 50% probability), and apply occlusions with random rectangles with up 5% of the size of the image.

### Data and materials

**Synthetic data:** We first evaluate registration performance on data collected from the phantom in [2]. For training, we assemble a database $F_{train}$ with manually adjusted spherical coordinate ranges $r_{min}=80$mm, $\delta r=10$mm, $r_{max}=300$mm, $\theta _{min}=108^\circ$, $\delta \theta =5^\circ$, $\theta _{max}=144^\circ$, and $\phi _{min}=216^\circ$, $\delta \phi =5^\circ$, $\phi _{max}=324^\circ$ and camera rotation angle ranges $\alpha _{min}=-45^\circ$, $\delta \alpha =15^\circ$, $\alpha _{max}=45^\circ$, $\beta _{min}=45^\circ$, $\delta \beta =5^\circ$, $\beta _{max}=45^\circ$, and $\gamma _{min}=-45^\circ$, $\delta \gamma =15^\circ$, $\gamma _{max}=120^\circ$, resulting in a total of $6.53\times 10^5$ poses.

**Table 1:** Spherical coordinate angular ranges used for database generation in eight clinical cases. $\#F$ refers to the number of instances in a database *F*

Case	[$\theta _{min}:\delta \theta :\theta _{max}$]	[$\phi _{min}:\delta \phi : \phi _{max}$]	$\#F_{train}$	$\#F_{test}$
1	[$100^{\circ }:10^{\circ }:140^{\circ }$]	[$40^{\circ }:10^{\circ }:110^{\circ }$]	3.56$\times 10^{5}$	2.86$\times 10^{6}$
2	[$100^{\circ }:10^{\circ }:140^{\circ }$]	[$60^{\circ }:10^{\circ }:130^{\circ }$]	4.17$\times 10^{5}$	3.13$\times 10^{6}$
3	[$80^{\circ }:10^{\circ }:120^{\circ }$]	[$80^{\circ }:10^{\circ }:140^{\circ }$]	3.49$\times 10^{5}$	2.68$\times 10^{6}$
4	[$120^{\circ }:10^{\circ }:145^{\circ }$]	[$60^{\circ }:10^{\circ }:160^{\circ }$]	2.26$\times 10^{5}$	1.75$\times 10^{6}$
5	[$110^{\circ }:10^{\circ }:180^{\circ }$]	[$20^{\circ }:10^{\circ }:120^{\circ }$]	5.24$\times 10^{5}$	2.01$\times 10^{6}$
6	[$100^{\circ }:10^{\circ }:150^{\circ }$]	[$60^{\circ }:10^{\circ }:140^{\circ }$]	3.14$\times 10^{5}$	2.37$\times 10^{6}$
7	[$90^{\circ }:10^{\circ }:130^{\circ }$]	[$70^{\circ }:10^{\circ }:150^{\circ }$]	6.18$\times 10^{5}$	4.69$\times 10^{6}$
8	[$220^{\circ }:10^{\circ }:250^{\circ }$]	[$260^{\circ }:10^{\circ }:340^{\circ }$]	2.86$\times 10^{5}$	2.11$\times 10^{6}$

For $F_{test}$, we infer a database with the same ranges but with a finer resolution of $\delta \alpha =5^\circ$, $\delta \beta =5^\circ$, $\delta \gamma =10^\circ$, with a final larger size of $7.10\times 10^6$ poses.

**Phantom data:** The training database and retrieval database are the same as the data we acquired in synthetic data. For validation, we collect real tracked video data from the physical phantom inside a laparoscopic trainer and perform registrations with $F_{test}$. We collect 21 video frames with a Viking (www.conmed.com) stereo laparoscope that is optically tracked by a Polaris Vega (www.ndi.com) and calibrated with a rig reported in [21].

Contours are manually segmented from each image, and a ground truth pose is obtained through a point-based registration of the phantom to the optical tracking space with the help of a tracked pointer [2]. The mean error of this registration on four picked landmarks does not surpass 3 mm.

**Clinical data:** In the third experiment, we test our method on retrospective data from eight laparoscopic surgery cases where a manual registration from CT to video was performed with the system reported in [1, 2]. Segmentations of the liver surface, liver vasculature, and lesions are obtained from an abdominal contrast-enhanced CT of the patient through a commercial service (www.visiblepatient.com). We use the same optically tracked system, but with a Storz (www.storz.com) stereo laparoscope and a Polaris Spectra (www.ndi.com) and perform the same calibration procedure as above. Contours were segmented on the video frames where the manual registration was achieved.

For all databases *F* generated for both training and inference, we set all camera rotation ranges as $\alpha _{min}=\beta _{min}=\gamma _{min}=-45^\circ$ and $\alpha _{max}=\beta _{max}=\gamma _{max}=45^\circ$ and spherical radial range as $r_{min}=80$mm, $\delta r=10$mm, $r_{max}=250$mm. For training databases, we set camera rotation steps to $10^\circ$, and for inference, we set them at a finer resolution of $5^\circ$. Spherical coordinate angular ranges are adjusted per patient and listed in Table 1 together with the number of rendered poses per database.

**Fig. 2:**
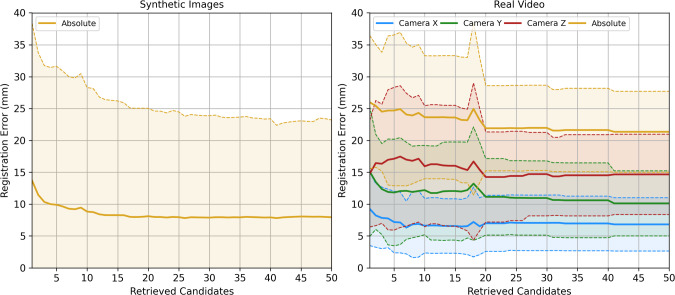
Registration error versus number of retrieved candidates on phantom experiments. Left shows mean and standard deviation of the registration error in mm across a sample 1000 synthetic views. Right shows mean and standard deviation of the registration error in mm across 21 laparoscopic video images of the physical phantom. Mean is represented by full lines, and standard deviation intervals are represented by dashed lines. On the right, blue, green, and red data series refer to the distance error projected across the camera x, y and z axes, respectively

**Fig. 3:**
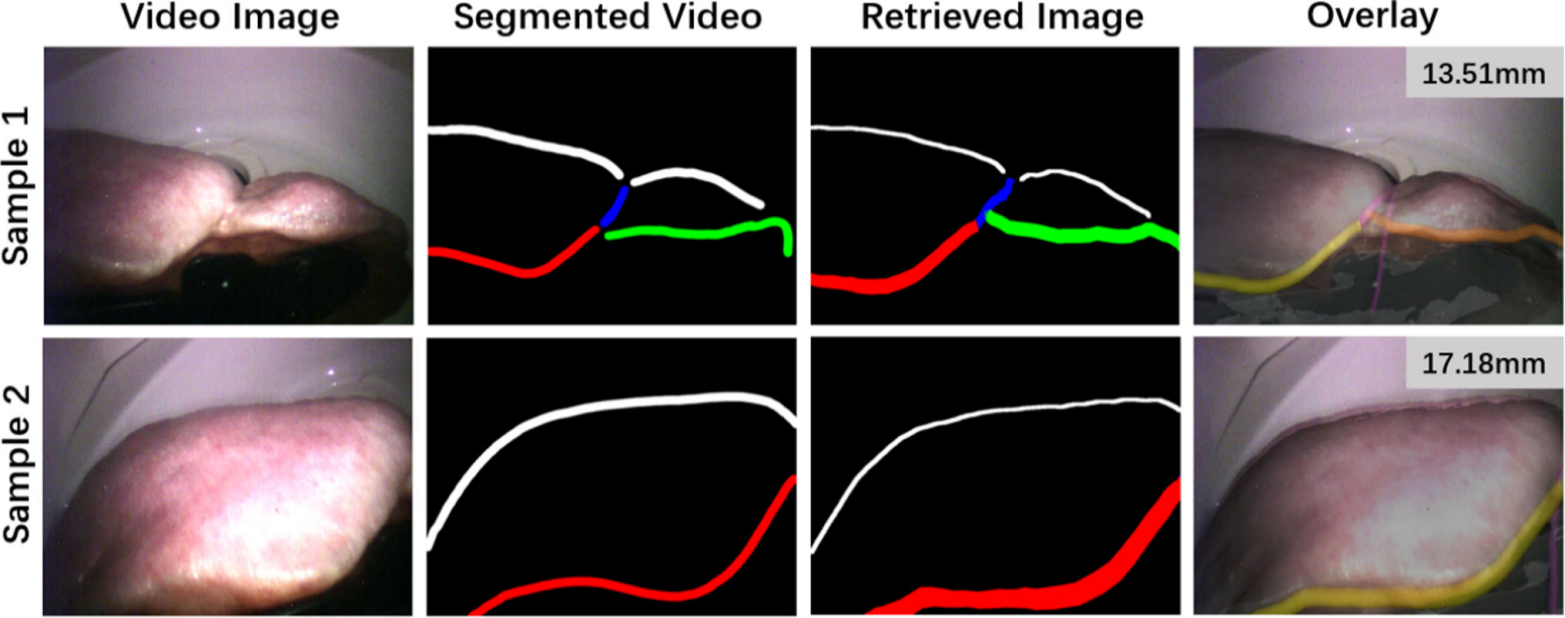
Visual results of two registration examples with real video images of the phantom. From left to right: original video, segmented contours in the video, contour map obtained through registration, and resulting overlay. On contour maps, white is silhouette, blue the falciform ligament, green the left ridge, and red the right ridge. On the overlay, purple is the falciform ligament, orange the left ridge, and yellow the right ridge

**Fig. 4:**
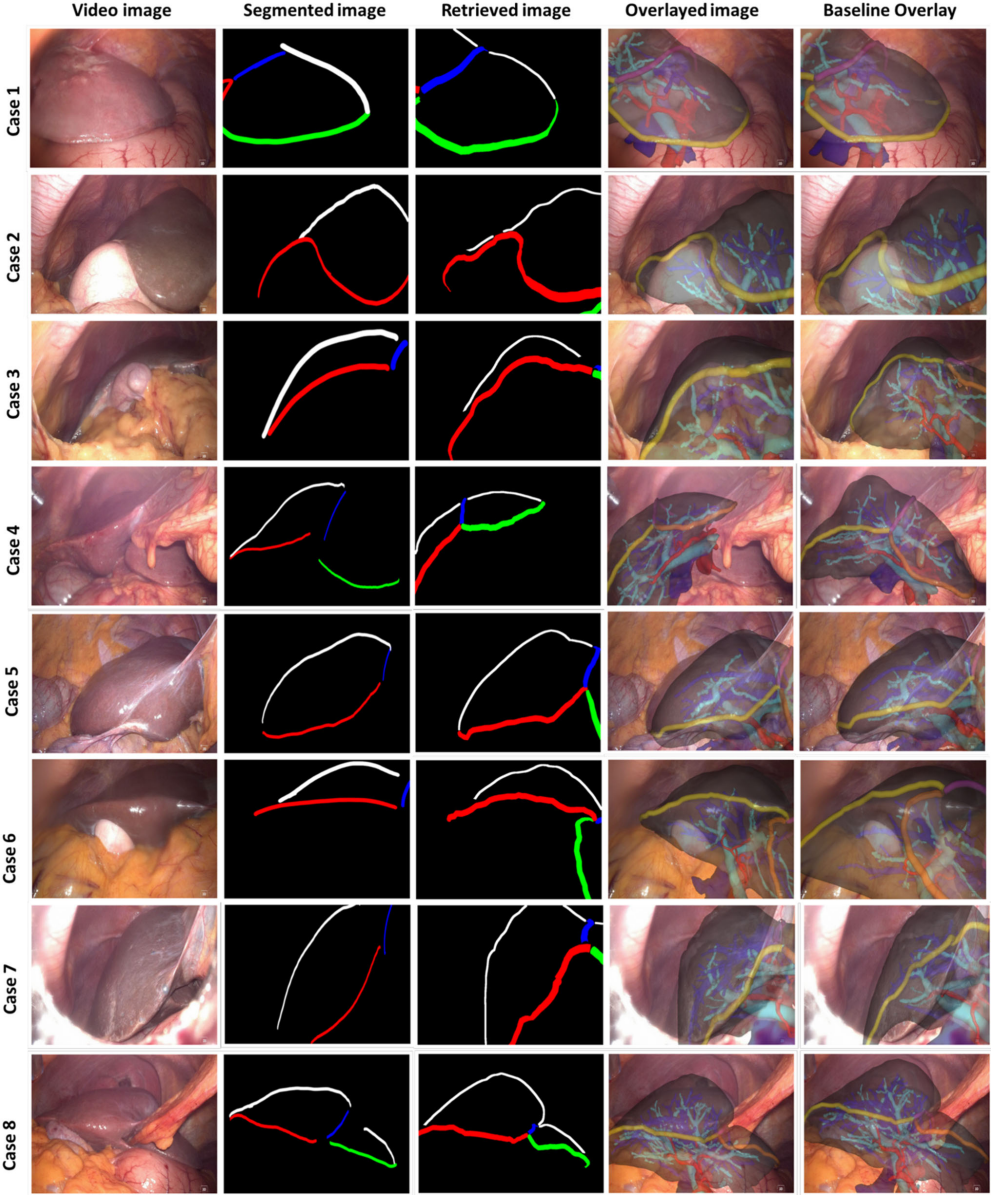
Visual results of registration for all eight surgical cases. From left to right: original video, segmented contours in the video, contour map obtained through registration, resulting overlay, and baseline overlay. Colour codes are the same as in Fig. 3. Baseline overlay shows hepatic veins in dark blue, portal vein in light blue, and arteries in red

### Phantom data evaluation

In the first experiment, we randomly extract 1000 samples of $F_{test}$ and apply the same random augmentations described in subsection 2.2.2. Then, we perform registrations by encoding the resulting images and using $F_{test}$ as target database. To measure registration error for any retrieved pose, we calculate the mean Euclidean distance between the liver surface points projected by the ground truth pose and the retrieved pose. For the experiment with real video data from the phantom, we repeat the same evaluation using the 21 tracked video images as input.

### Clinical data evaluation

To evaluate performance on each patient, we select the frame where a manual registration was obtained during surgery and visually compare its resulting overlay to the one obtained with DH. We choose to use this qualitative comparison as it is not possible to obtain a reliable ground truth registration for a quantitative error evaluation. Since the live surgery overlays were obtained with the assistance of surgeons, they hold enough value for a qualitative visual analysis. Furthermore, the main objective of our method is to replace this manual process.

## Results

### Phantom experiments

Mean registration accuracies for 1000 synthetic views with the phantom surface are presented on the left side of Fig. 2. With increasing *k* candidates, registration error decreases until *k*=20 to 8.0mm±15.3 and then stabilises.

Registration error results for the 21 real video images of the phantom are shown on the right side of Fig. 2. As expected, the higher absolute errors (yellow series) result in higher errors than in the synthetic case. However, the same trend is followed with *k*, but with a different value of convergence $k=20$ resulting in a mean error of 21.9mm±6.7. To better understand the source of these higher errors, we project this error across the three camera axes in three additional data series. Noticeably, higher errors are observed across the camera viewing direction (z, red series), with a mean error of 14.2mm±7.1.

Visual results of this registration with *k*=50 are exemplified in Fig. 3. In each row, from left to right we present the input video image, the manually segmented contours, the retrieved contours, and resulting AR overlay. Although errors above 10 mm are shown, there is a visual agreement between the obtained overlay and the video image in both the case where all contours are seen (Sample 1) and the case where only the right lobe is captured (Sample 2). Other examples are provided in the supplementary material.

### Clinical data experiments

Registration results for the eight patient cases using $k=50$ are displayed in Fig. 4. In each row, we present the same set of results as in Fig. 3, but add the AR overlay obtained manually during surgery to the right. In five out of eight cases (Cases 1, 2, 5, 7, and 8), the automated DH alignment shows an agreement with the manual solution. For Cases 1, 2, and 7, it is noticeable that the DH approach actually provides an overlay that better aligns the ridge and silhouette contours. This is also observed on the Case 6, where a seemingly more accurate alignment is seen on the right lobe. Case 3 shows an example where both alignments have the correct orientation, but wrong depth. For Case 4, both approaches result in an inaccurately aligned overlay, with the manual alignment performing better. This case illustrates the challenge of rigid registration in the presence of a large deformed view of the liver with ligament occlusion.

## Discussion and conclusion

Phantom experiment results quantitatively demonstrate the feasibility of DH and Hausdorff ranking as a solution for the video to CT registration problem. Synthetic experiments show that including more candidates in the registration reduces registration error only up to a point. The large deviations of 15 mm in this experiment are explained by the coarseness of the database—the registration error is either 0 mm when the original pose is retrieved, or a higher value if an adjacent pose is found. Since positions are parameterised spherically, poses simulated further away from the liver become more separated in space.

The same deviation effects occurred in the video frames of the phantom. However, the mean errors are higher as the ground truth is influenced by errors in the point-based registration, optical tracking, and laparoscope calibration. Errors across each axis indicate that registration was less accurate across the camera viewing direction—this is likely to be explained by contour thickness ambiguities in this axis. In the future, we intend to integrate depth cues into the segmented liver contours to create more unique hash codes [22]. Regardless, our visual results (Fig. 3) show a good agreement with the real scene, suggesting our method is successful in a rigid scenario.

Our results on clinical data are encouraging as only one case resulted in a completely invalid alignment. Even though it is not possible to have a registration error evaluation, visual observation suggests that DH can replace the manual initialisation of registration in surgery. Furthermore, in four out of eight cases, DH estimated a potentially more accurate overlay than the manual alignments. This is of particular importance considering how disruptive these manual interactions currently are—in these eight cases, manual alignment was obtained on a single frame using mouse click and scrolling controls that took on average 2:30 min. With a non-optimised implementation, our method requires around 4.5 s to register, where the main computation time comes from the weighted Hausdorff distance calculation that is not parallelised and requires re-rendering of 2D images. This opens up the possibility of quickly obtaining an initial rigid alignment in any partial view of the liver throughout surgery. Our visual results support this idea, as six alignments were obtained on a partial view with a single lobe.

Poorer results occur mostly in larger views where a rigid method cannot compensate for the observed global deformation (Case 4). Best results are obtained in partial views where there is no ligament occlusion and local rigidity is more valid. To improve performance in all scenarios, future work will focus on adjusting our pre-operative simulation and DH training to include non-rigid parameters and occlusions due to the falciform ligament. Although we have used manual segmentation in our evaluation, given sufficient data there are several options to achieve an automatic result, either using CNN models [13] or the more recent foundation models [22]. We have not been able to obtain registration accuracy results on our clinical data as the manual alignments performed in surgery did not set a reliable reference. In future work, we aim to perform this quantitative evaluation on existing benchmarks [3].

Compared to existing methods, our method currently requires a pre-operative time expense of 20 h for patient-specific model training and database generation. Although this expense is acceptable within the current clinical workflow, in the future we aim to speed up this process by training a general hashing model on multiple patients, keeping the database generation as the only patient-specific step.

Overall, we have introduced the first DH approach for registration of 2D video to 3D CT in laparoscopic liver surgery to enable AR. The method is self-supervised, does not require real data for training, has the potential of replacing a manual rigid registration in surgery, and enables the clinical translation of AR. Further refinements will be introduced to account for insufflation induced deformations and to automate segmentation.

## Supplementary Information

Below is the link to the electronic supplementary material.

Supplementary file 1 (pdf 659 KB)
