# Correction: Deep hashing for global registration of preoperative CT and video images for laparoscopic liver surgery

**DOI:** 10.1007/s11548-025-03488-w

**Published:** 2025-08-31

**Authors:** Hanyuan Zhang, Sandun Bulathsinhala, Brian R. Davidson, Matthew J. Clarkson, João Ramalhinho

**Affiliations:** 1 Department of Medical Physics and Biomedical Engineering, UCL Hawkes Institute, UCL, Oxford, UK; 2 Division of Surgery and Interventional Science, UCL, Oxford, UK

**Correction to: International Journal of Computer Assisted Radiology and Surgery (2025) 20:1461–1469** 10.1007/s11548-025-03418-w

In the original version of this article, sixth sentence under the section “Pose generation and rendering”, which previously read as

To sample different orientations, we set three ranges for Euler angles (*α* = *α*_*min*_: *δα*: *α*_*max*_, *β = wesetthreeranges f or Eulerangles(α = αmin: δα: αmax, β = δmin: δβ: βmax and γ = γ min: δγ: γ max), min: δβ: βmaxandγ = γ min: δγ: γ max*), and apply all possible combinations of Euler rotations at every sampled position in space.

but should have read

To sample different orientations, we set three ranges for Euler angles ($\alpha = \{ \alpha_{min}{:}\,\delta \alpha {:}\,\alpha_{max} \} ,\beta = \{ \beta_{min} {:}\,\delta \beta {:}\,\beta_{max} \}$ and $\gamma = \{ \gamma_{min} {:}\,\delta \gamma{:}\,\gamma_{max} \}$), and apply all possible combinations of Euler rotations at every sampled position in space.

The original article has been corrected.

