# Supplementary material for: Deep hashing for global registration of preoperative CT and video images for laparoscopic liver surgery
Source: Int J Comput Assist Radiol Surg. 2025 May 23;20(7):1461–9. doi: 10.1007/s11548-025-03418-w (PMC12226707; doi:10.1007/s11548-025-03418-w)
Supplement: Supplementary file 1 — (pdf 659 KB) [file 11548_2025_3418_MOESM1_ESM.pdf]

Sample1:

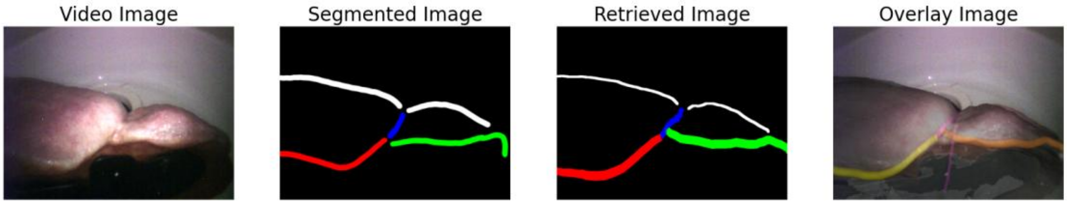

Mean distances(mm):13.51

Sample2:

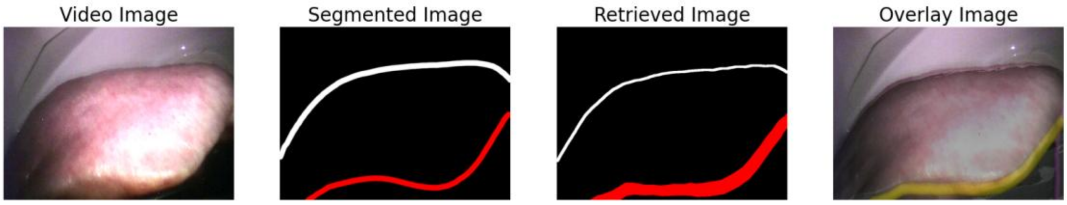

Mean distances(mm):17.19

Others not presented in paper:

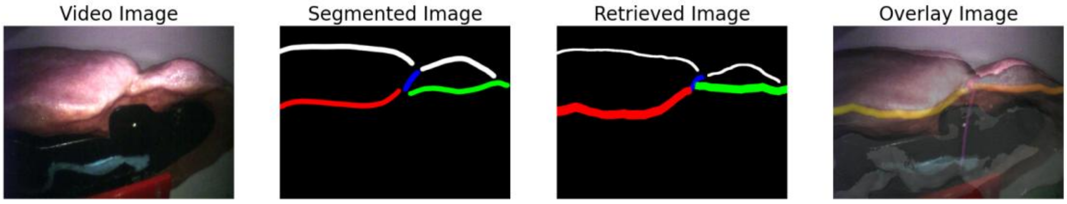

Mean distances(mm):14.11

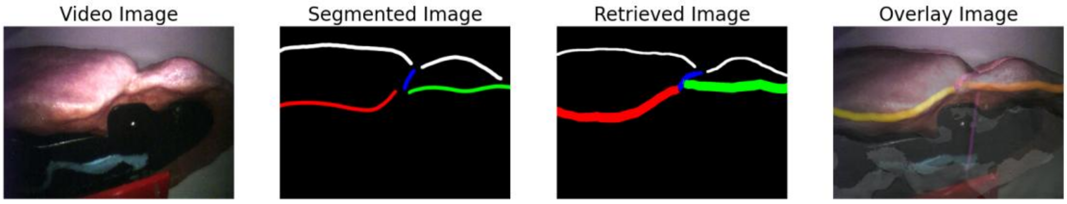

Mean distances(mm):23.11

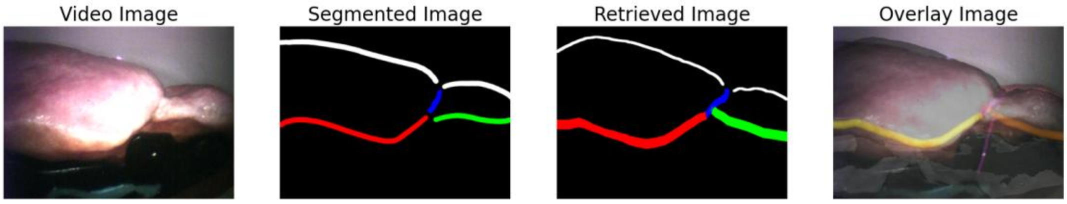

Mean distances(mm):28.21

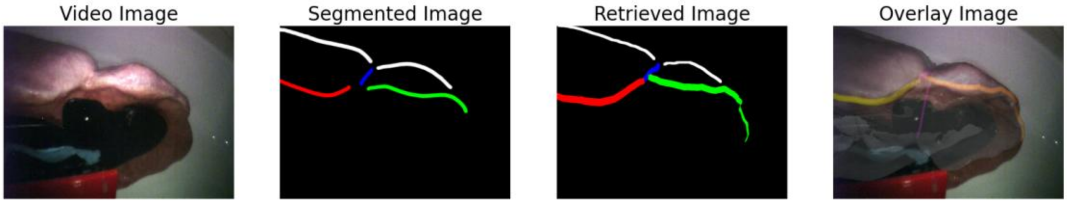

Mean distances(mm):21.3

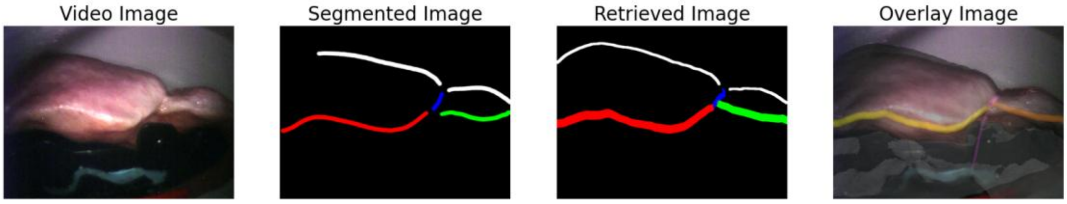

Mean distances(mm):20.11

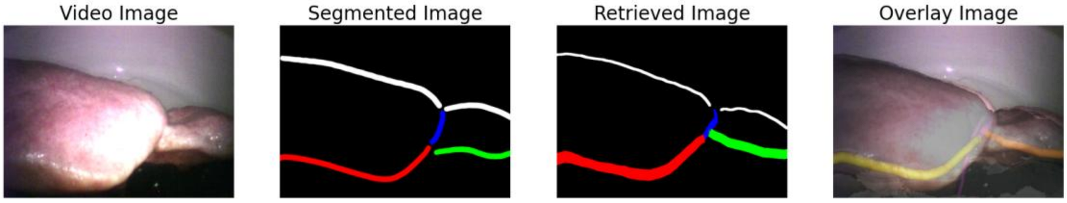

Mean distances(mm):14.9

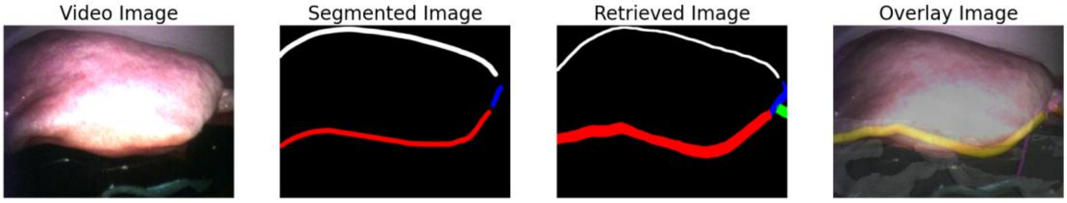

Mean distances(mm):18.15

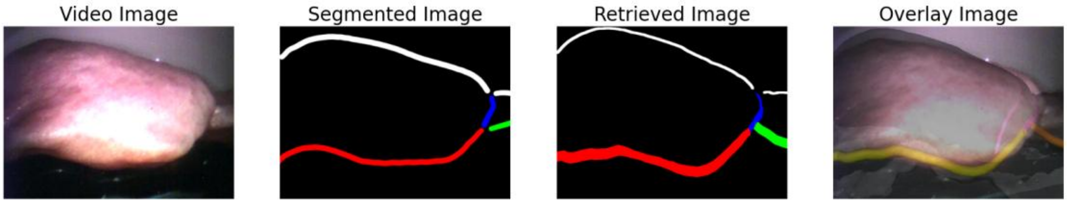

Mean distances(mm):16.11

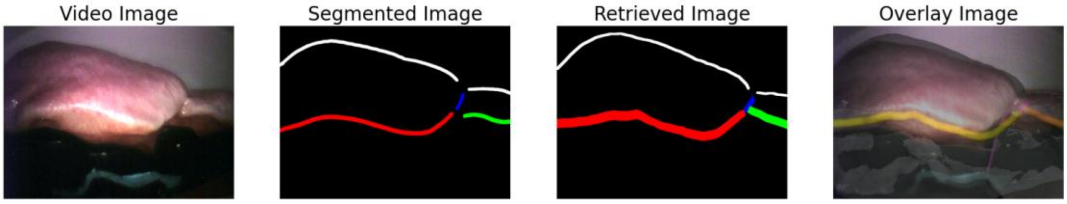

Mean distances(mm):13.39

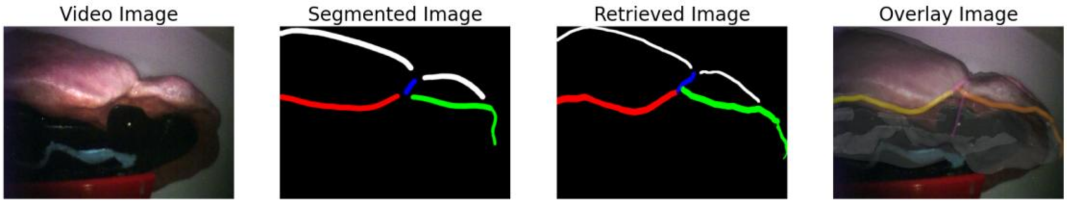

Mean distances(mm):31.27

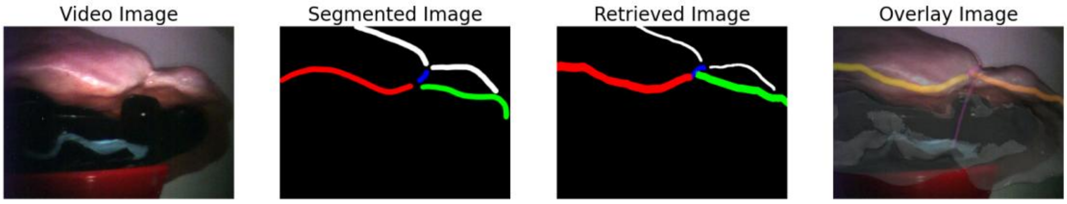

Mean distances(mm):24.04

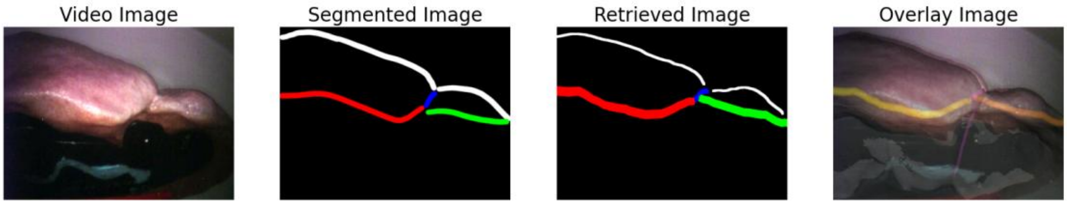

Mean distances(mm):16.72

Video Image | Segmented Image | Retrieved Image | Overlay Image

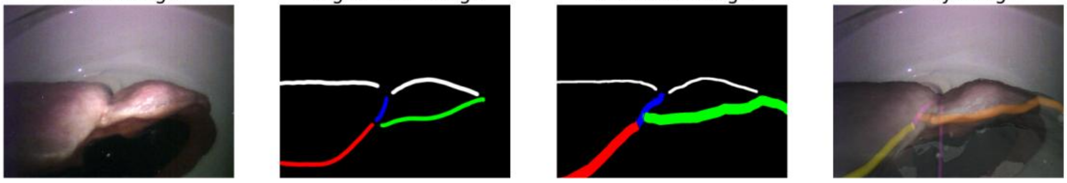

Mean distances(mm):25.72

Video Image | Segmented Image | Retrieved Image | Overlay Image

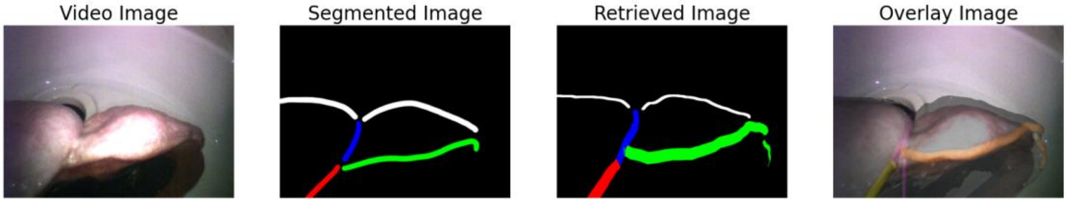

Mean distances(mm):18.95

Video Image | Segmented Image | Retrieved Image | Overlay Image

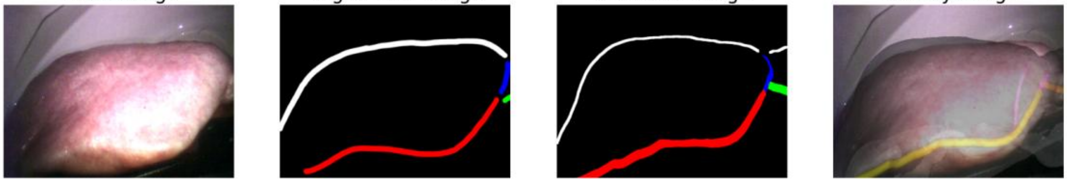

Mean distances(mm):35.52

Video Image | Segmented Image | Retrieved Image | Overlay Image

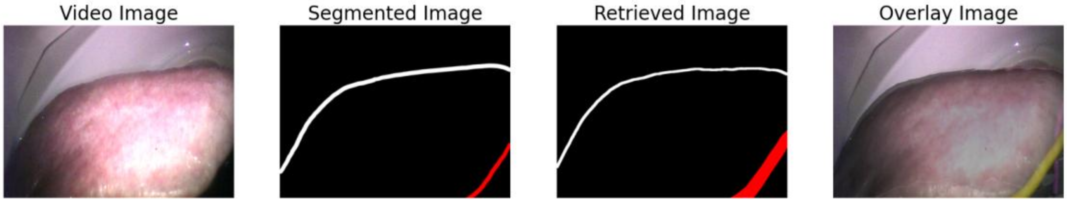

Mean distances(mm):27.1

Video Image | Segmented Image | Retrieved Image | Overlay Image

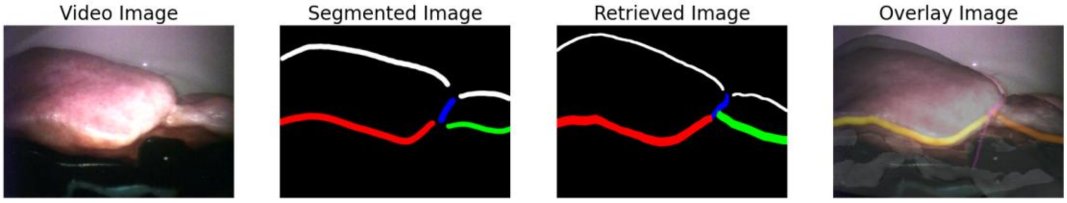

Mean distances(mm):14.9

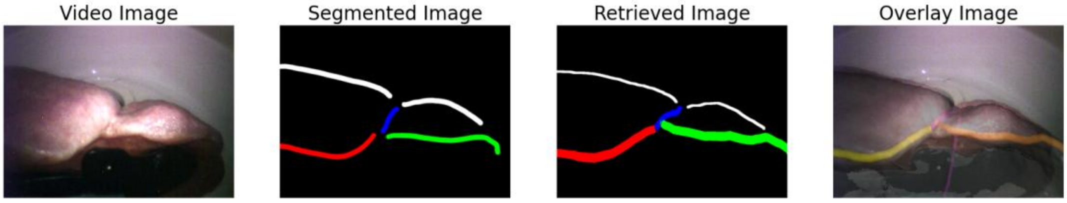

Mean distances(mm):22.95

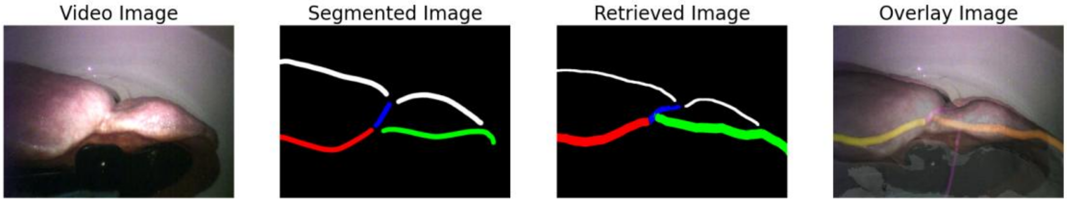

Mean distances(mm):31.03
